# Two-stage surgical correction of L4/5 non-union using ALIF and endoscopic TLIF cage removal: a case report

**DOI:** 10.1093/jscr/rjaf385

**Published:** 2025-06-08

**Authors:** Ralph J Mobbs

**Affiliations:** Faculty of Medicine and Health, University of New South Wales, Barker St., Randwick, 2031, NSW, Australia; NeuroSpine Surgery Research Group, Barker St., Randwick, 2031, NSW, Australia; NeuroSpineClinic, Suite 7, Level 7, Prince of Wales Private Hospital, 1 Barker St., Randwick, 2031, NSW, Australia; WAGAR. Wearables and Gait Assessment Research Group, 203 Avoca St., Randwick, 2031, NSW, Australia

**Keywords:** ALIF, TLIF cage removal, spinal pseudarthrosis, endoscopic spine surgery, lumbar fusion failure, minimally invasive revision

## Abstract

Anterior lumbar interbody fusion (ALIF) offers a viable solution for failed posterior fusion procedures, including non-union following transforaminal lumbar interbody fusion (TLIF). This case report presents a novel two-stage corrective approach involving ALIF followed by endoscopic removal of a failed TLIF cage in a patient with L4/5 pseudarthrosis and persistent symptoms. Stage one involved placement of an ALIF cage with integral fixation, while stage two comprised minimally invasive endoscopic decompression and TLIF cage retrieval. The procedure was completed successfully without complications, resulting in improved neurological status, segmental stabilization, and symptomatic relief. This case illustrates a novel and effective surgical strategy for managing interbody non-union and highlights the role of endoscopic techniques in revision spine surgery.

## Introduction

Lumbar interbody fusion (Fig. 6) has become a foundational technique in the surgical management of degenerative disc disease, segmental instability, and other spinal pathologies requiring structural stabilization and neural decompression [1]. However, despite advancements in surgical instrumentation, biological adjuncts, and operative techniques, pseudarthrosis (non-union) remains a significant and challenging complication following transforaminal lumbar interbody fusion (TLIF) procedures. Non-union can result in persistent axial back pain, recurrent radiculopathy, mechanical instability, and diminished quality of life—often necessitating complex revision surgery [2]. Conventionally, revision strategies have centered on open posterior re-exploration and hardware revision, approaches associated with increased soft tissue disruption, higher blood loss, and longer recovery times. In response to these limitations, there has been increasing interest in less invasive, anatomically favorable alternatives, particularly through anterior and endoscopic spinal approaches [3].

**Figure 1:**
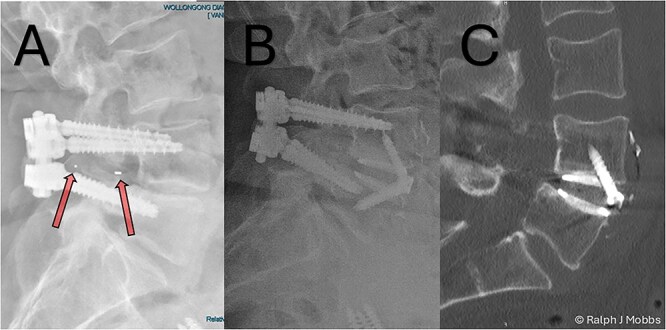
(A) Initial presentation with TLIF cage migration posteriorly into the spinal canal and paraspinal muscles. (B) Stage 1 and 2 completion, including revision fusion using an ALIF cage (Redmond Ti/PEEK, A-spine, Taiwan) and TLIF cage removal. (C) CT scan post TLIF removal and disc height restoration with ALIF cage.

This case report illustrates a novel two-stage revision strategy for symptomatic L4/5 pseudarthrosis (Fig. 1A), integrating anterior lumbar interbody fusion (ALIF) with posterior endoscopic cage retrieval and decompression. The initial ALIF procedure (Fig. 1B) enabled restoration of segmental lordosis, disc height, and immediate biomechanical stability, while also facilitating distraction of scarred posterior elements. This was followed by a targeted, minimally invasive endoscopic procedure to remove the failed TLIF cage and decompress neural elements (Video 1), thereby avoiding the morbidity of an open posterior revision.

The aim of this report is to present an innovative, hybrid surgical approach for addressing complex interbody fusion failure. By combining the mechanical advantages of anterior reconstruction with the precision and reduced invasiveness of endoscopic techniques, this strategy seeks to minimize perioperative morbidity while optimizing clinical outcomes. Through detailed operative description and visual documentation, this report contributes to the evolving landscape of revision spine surgery and supports the integration of multidisciplinary and minimally invasive solutions in complex spinal pathology.

## Case report

### Patient background

A 64-year-old female with a history of a TLIF at L4/5, performed 18 months prior to presentation to the current surgeon, presented with persistent low back pain and radiculopathy. Imaging demonstrated non-union at the TLIF site with ongoing foraminal stenosis and segmental instability (Fig. 1A). Multidisciplinary review determined that a revision fusion was the initial focus as mechanical back pain related to non-union was the primary complaint. Following the planned stage 1 intervention, removal of the migrated TLIF cage was determined necessary as the device was serving no purpose and likely causing further symptoms. Therefore, a two-stage revision strategy was planned.

#### Stage one: anterior lumbar interbody fusion

**Figure 2:**
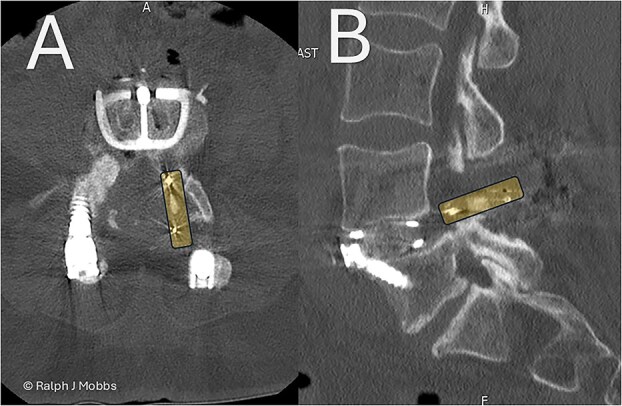
Day 1 postop ALIF. The PEEK cage can be seen in the canal and paraspinal regions. (A) Axial sequence. (B) Parasagittal sequence.

Following standard preoperative preparation, the patient underwent ALIF at L4/5 (Fig. 6). A standard retroperitoneal approach and anterior discectomy were performed [4], with the disc space prepared. Note was made that no graft was within the interbody space, nor any evidence of arthrodesis. A Redmond Ti/PEEK ALIF cage (A-Spine, Taiwan) 14 mm, 12° lordotic cage was inserted with three integral 30 mm screws for segmental fixation (Fig. 1B and C). The ALIF device and interbody space were packed with allograft and autograft material. The construct was confirmed with intraoperative X-ray imaging, and postop CT scan the following day (Fig. 2). Estimated blood loss was minimal (~15 cc), with no intraoperative complications.

#### Stage two: endoscopic TLIF cage removal and Rhizolysis

**Figure 3:**
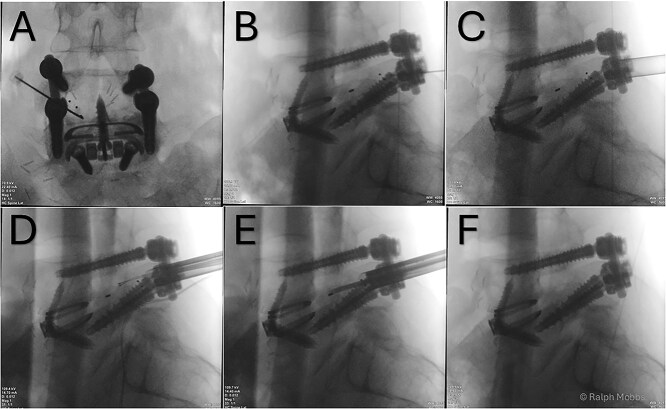
(A) AP X-ray. Initial needle localization over the TLIF cage. (B) Lateral X-ray with needle positioned on posterior aspect of TLIF cage. (C) Working channel positioned immediately posterior to cage. (D) RF diathermy to dissect around cage (see Video 1). (E) Dissection around cage using multiple instruments. (F) Post removal of cage, with no TLIF device evident.

**Figure 4:**
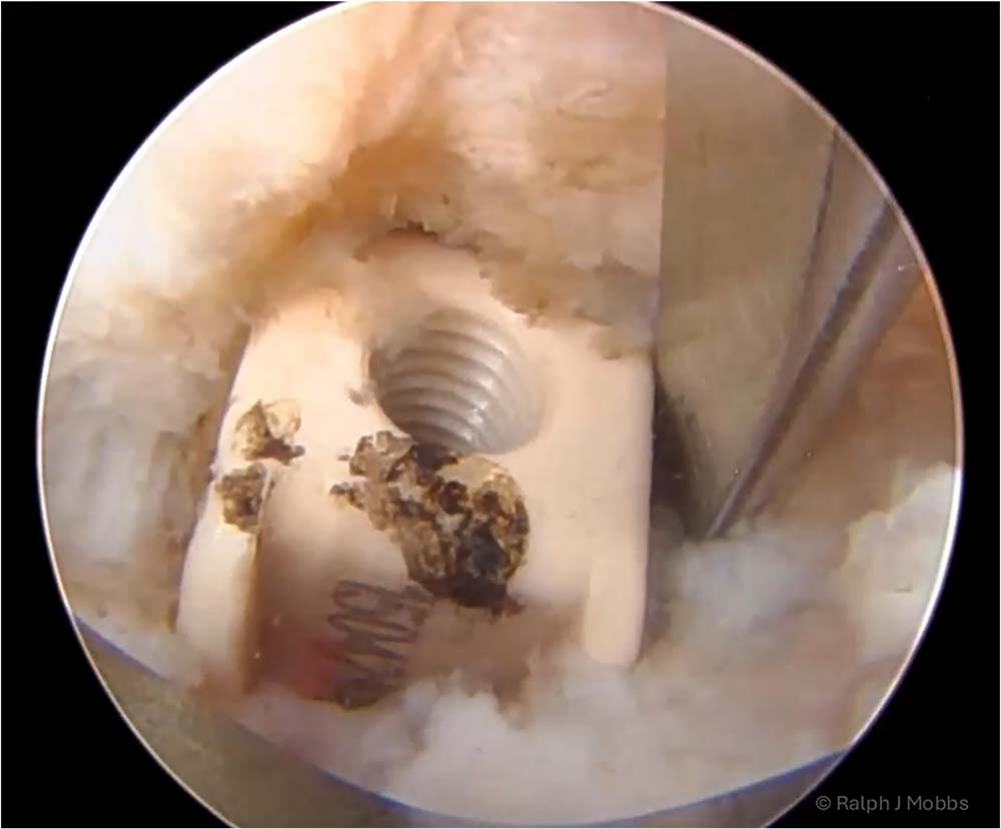
Endoscopic view of the TLIF cage in scar with dissection around the device prior to removal of the migrated TLIF cage.

**Figure 5:**
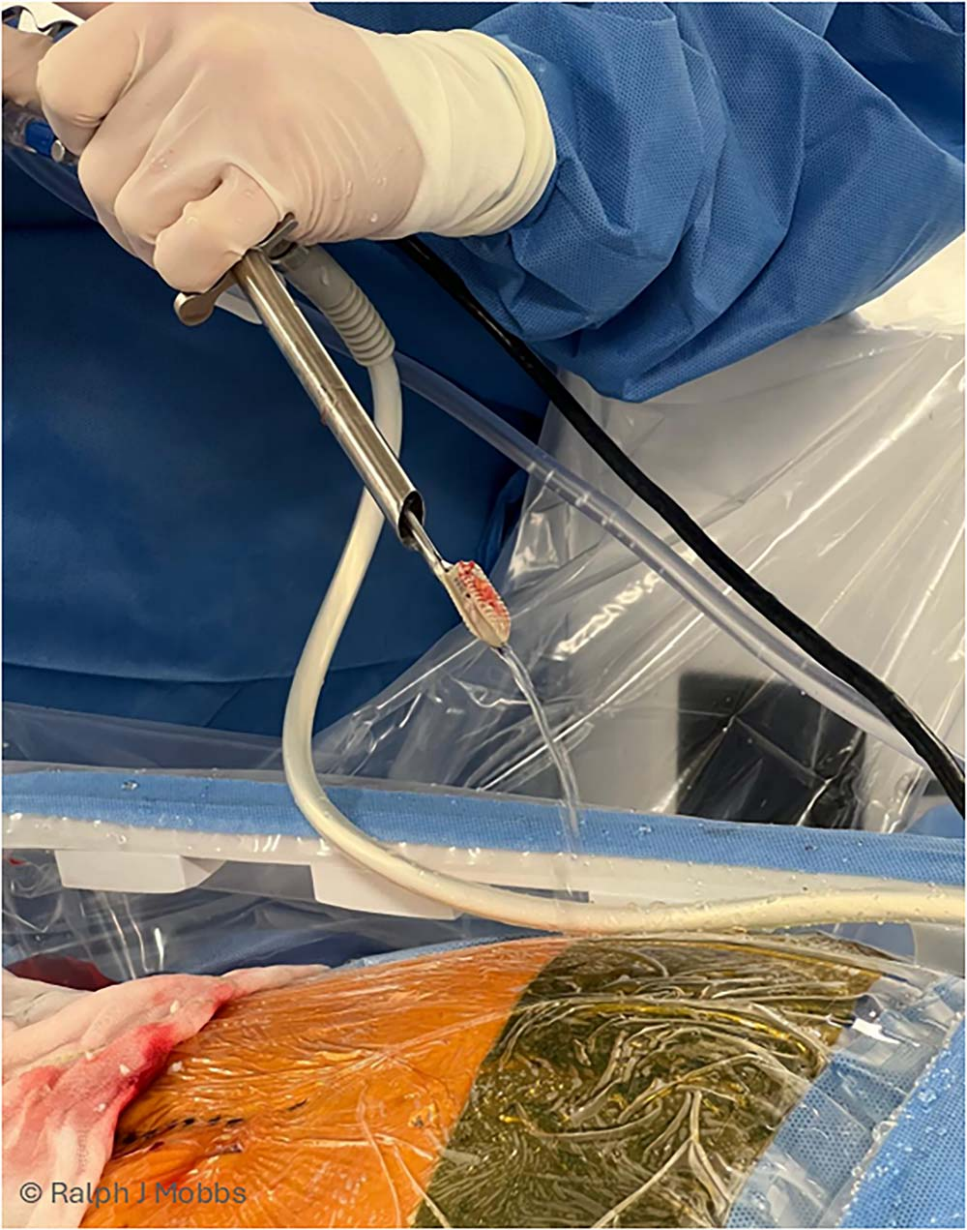
Immediate post removal with the endoscope, working channel, grasping tool (endoscopic ligament cutter), and TLIF cage removed as one (see Video 1 for maneuver).

After an adequate interval recovery of 3 days, the patient underwent the second stage of minimally invasive removal of the TLIF cage via an endoscopic approach. The Elliquence Stenosis endoscopic system was used (Elliquence, USA) for TLIF retrieval. Under fluoroscopic guidance, the TLIF cage was identified with a dilator positioned directly superficial to the cage, then a 10 mm working channel positioned to insert the endoscope (Fig. 3). Dissection of the TLIF cage (Fig. 4), mobilization, and retrieval (Fig. 5) was achieved with relative ease (Video 1). Using constant irrigation through the endoscope, this assisted with both hemostasis and hydrodissection of the tissue planes around the cage to aid removal. Hemostasis was achieved, and the wound was closed in standard fashion.

**Figure 6:**
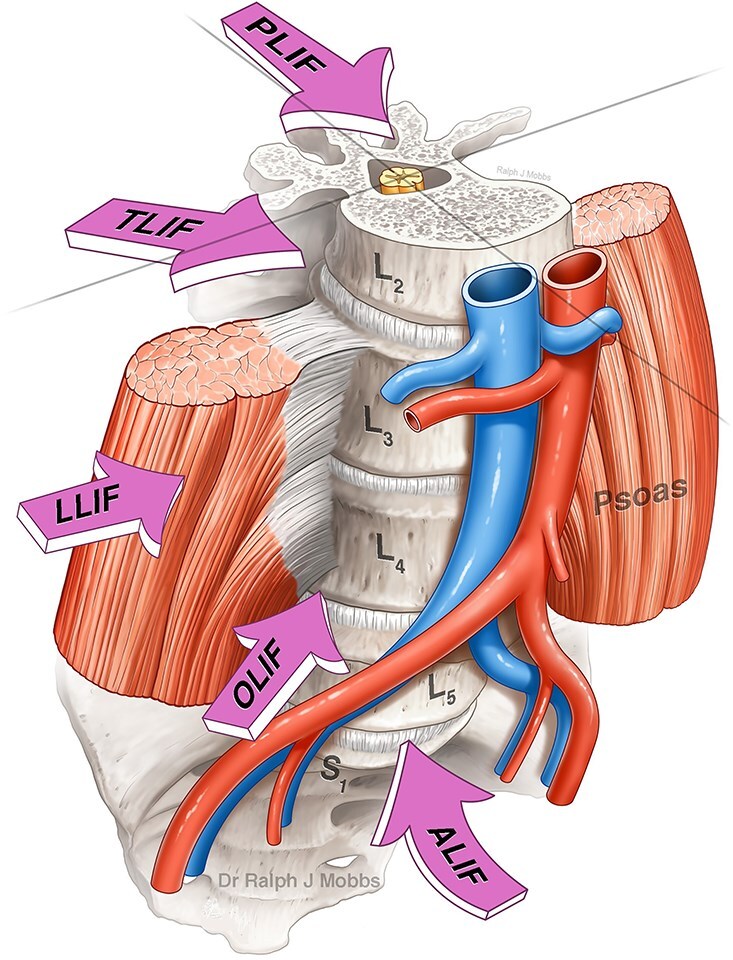
Lumbar interbody fusion (LIF) techniques (with permission of Mobbs *et al.*, JSS).

## Discussion

This case highlights an innovative sequential revision strategy for TLIF non-union, integrating anterior structural reinforcement through ALIF and minimally invasive posterior endoscopic cage removal and decompression [5, 6]. This hybrid approach leverages the biomechanical advantages of anterior reconstruction while minimizing the morbidity associated with traditional posterior re-entry.

The ALIF procedure provided immediate segmental stabilization, restored disc height, and improved sagittal alignment, while also contributing to the indirect decompression of neural elements by elevating the disc space. Notably, the increased disc height achieved through ALIF appeared to facilitate posterior cage retrieval by exerting distraction on the surrounding scar tissue, thereby easing dissection through previously adherent tissue planes.

Conversely, endoscopic posterior cage removal enabled targeted decompression and hardware retrieval without the extensive tissue disruption inherent to open posterior revision surgery. This technique offers a promising alternative that reduces intraoperative morbidity, preserves soft tissue integrity, and may shorten recovery time.

Key advantages of this approach include:

(i) Restoration of segmental lordosis and disc height via anterior reconstruction (ALIF).

(ii) Minimally invasive posterior decompression and cage removal through endoscopy.

(iii) Reduced intraoperative blood loss and soft tissue trauma.

(iv) Avoidance of extensive and potentially destabilizing posterior reoperation.

Despite these benefits, this strategy necessitates a two-stage operative plan and relies on the surgeon’s expertise with endoscopic spinal techniques—factors that may limit its broader adoption. However, as surgical technology and minimally invasive techniques continue to evolve, this dual-modality approach presents a compelling template for managing complex revision scenarios in lumbar interbody fusion failure.

Moreover, the integration of artificial intelligence in preoperative planning and intraoperative navigation holds considerable promise for enhancing decision-making in complex spinal reconstruction [7]. As such, the future of revision spine surgery will likely incorporate synergistic combinations of anterior and posterior techniques, underpinned by data-driven, patient-specific strategies to optimize outcomes.

## Conclusion

This case demonstrates a novel and effective revision strategy for TLIF non-union, combining ALIF with endoscopic posterior cage removal. This approach offers enhanced segmental stability, restoration of disc height, and reduced surgical morbidity through minimally invasive techniques. While promising, broader validation through case series and comparative studies is needed to establish standardized protocols for complex spinal revision surgery.

## Supplementary Material

Video_1_ALIF_TLIF_Endo_rjaf385
